# Optimization of Membrane Electrode Assembly of PEM Fuel Cell by Response Surface Method

**DOI:** 10.3390/molecules24173097

**Published:** 2019-08-26

**Authors:** Rohit K. S. S. Vuppala, Benitta A. Chaedir, Lishuai Jiang, Lianjun Chen, Muhammad Aziz, Agus P. Sasmito

**Affiliations:** 1 Department of Mining and Materials Engineering, McGill University, 3450 University, Frank Dawson Adams Bldg., Montreal, QC H3A2A7, Canada; 2 Mechanical Engineering Department, National Institute of Technology, Tiruchirapalli 620 015, India; 3 State Key Laboratory of Mining Disaster Prevention and Control, Shandong University of Science and Technology, Qingdao 266590, China; 4 Institute of Industrial Science, The University of Tokyo, 4-6-1 Komaba, Meguro-ku, Tokyo 153-8505, Japan

**Keywords:** PEM fuel cell, membrane electrode assembly (MEA), response surface method, computational fuel cell dynamics

## Abstract

The membrane electrode assembly (MEA) plays an important role in the proton exchange membrane fuel cell (PEMFC) performance. Typically, the structure comprises of a polymer electrolyte membrane sandwiched by agglomerate catalyst layers at the anode and cathode. Optimization of various parameters in the design of MEA is, thus, essential for reducing cost and material usage, while improving cell performance. In this paper, optimization of MEA is performed using a validated two-phase PEMFC numerical model. Key MEA parameters affecting the performance of a single PEMFC are determined from sensitivity analysis and are optimized using the response surface method (RSM). The optimization is carried out at two different operating voltages. The results show that membrane thickness and membrane protonic conductivity coefficient are the most significant parameters influencing cell performance. Notably, at higher voltage (0.8 V per cell), the current density can be improved by up to 40% while, at a lower voltage (0.6 V per cell), the current density may be doubled. The results presented can be of importance for fuel cell engineers to improve the stack performance and expedite the commercialization.

## 1. Introduction

As a clean energy device, a proton exchange membrane (PEM) fuel cell is a promising power-generating technology that has received increasing attention over the last decade. Fuel cell is an electrochemical device that converts chemical energy into electrical energy. Due to its high energy-conversion efficiency and zero-emission potential, the fuel cell is considered as the best power-generating device, especially in transportation applications. Among the different kinds of fuel cells, PEM fuel cell (PEMFC) offers desirable features, such as low operational temperature and high-power density, which makes it the most promising alternative technology for power production.

In order for the fuel cell technology to be competitive with conventional power systems, some challenges associated with it, including reliability, longevity, and cost, must be overcome. A better understanding of the system is required to achieve the ideal price-performance balance. Studies have been carried out to characterize the behavior of the PEMFC system as affected by different parameters. Wang et al. [1] conducted parametric experiments to study the effect of various operating parameters on the performance of a single PEMFC and used the results to validate the 3D model they developed. The parameters studied were pressure, fuel cell temperature, and anode and cathode relative humidity. It was observed that, generally, increasing the fuel cell temperature and pressure increases its performance, except when the temperature is higher than the gas stream humidification temperatures, especially at a low current density region. Cathode humidification temperature was found to have no significant impact on fuel cell performance, while increasing anode humidification temperature increases performance at the low current density region. These results are in accordance with the results obtained by Ferng et al. [2] and Amirinejad et al. [3] who concluded that operation at higher pressure and elevated temperature can improve the electrode kinetic performance and increase the ionic conductivity in membrane and electrodes, which results in high power density in the PEMFC system. Santarelli and Torchio [4] experimentally analyzed the performance of a PEMFC by varying cell temperature, anode and cathode flow temperatures in both saturation and dry conditions, and reactant pressure. The results showed that, in addition to cell temperature, an increase in reactant saturation temperature also leads to a better performance and the best improvements due to a pressure increase are observed when both anode and cathode are humidified. Yan et al. [5] extended the study of the effects of operating conditions to PEMFC with interdigitated flow field. Nafion-based PEM fuel cell performance analysis with various reactant humidification levels, which varied from no external humidification to a fully saturated anode and cathode, was carried out by Williams et al. [6]. Klika et al. [7] have developed a thermodynamically-consistent model based on polynomial functions to study the behavior of water sorption in Nafion membranes. A three-dimensional multiphase numerical model was developed by Fan et al. [8] to study the PEMFC performance at low external humidification. It was found that the dependency on external humidification of a PEMFC can be cut down at high current density, due to the water produced in the cathode catalyst layer that is sufficient to be employed to humidify both the cathode and anode polymer electrolyte.

In addition to operating conditions, there are other parameters affecting the fuel cell performance. Bayrakçeken et al. [9] found that membrane thickness, hot-pressing conditions of the gas diffusion layer (GDL), and the Teflon to carbon ratio in the GDL, are also significant parameters to provide good PEMFC performance. The study showed that thinner membrane thickness and higher Teflon:carbon ratio in the GDLs give better performances. Jiang et al. [10] implemented an effective “elementary effect” (EE) method based on Monte Carlo random experiments to analyze 22 uncertain parameters involved in their two-phase 1D analytical PEMFC model. Among all of the parameters, membrane thickness and volume fractions were found to be the most important factors influencing the cell performance. The effect of catalyst layer microstructure was recently investigated numerically by Carcadea et al. [11]. A CFD model was used to study the behavior of a PEMFC as a function of Pt loading, Pt particle radius, ionomer volume fraction, and carbon support, and to establish the optimum range of these parameters. It was observed that increasing the ionomer volume fraction in the catalyst layer (CL) leads to better performance due to the fact that the ionomer acts as a network for the mass and charge transport. Moreover, higher Pt loading and a lower particle radius are recommended to achieve better PEMFC performance. Lee et al. [12] investigated the performance improvement of a PEMFC as a function of gas diffusion layer porosity and impregnation of the Nafion solution.

The previously mentioned studies confirm the significance of various parameters on the operation of the PEMFC. It is, therefore, crucial to select the optimum values in order to achieve a high-performance fuel cell. Efforts have been made by researchers toward the optimization of critical parameters influencing the PEMFC operation using different approaches. Salva et al. [13] developed a one-dimensional analytical model and used it to obtain the operating conditions under which a single PEMFC provides the maximum power output for different current intensities. The optimization was carried out for every value of current intensity by solving the parametric table consisting of all possible combinations obtained from modifying the stoichiometry in the cathode and anode, relative humidity in the anode and cathode, and the operating temperature, while keeping the pressure constant. Wu et al. [14] employed a multi-resolution approach and the radial basis function (RBF) surrogate model for simulation and optimization of operating conditions for hydrogen polymer electrolyte fuel cells. Zervas et al. [15] performed a phosphoric acid fuel cell (PAFC) system optimization study based on meta-models that were derived by applying the linear regression and the RBF neural network methodology on the results produced by a CFD model. The optimization of different operating and design parameters on PEMFC using the Taguchi method was performed by Karthikeyan et al. [16], Solehati et al. [17], and Sasmito et al. [18]. Grujicic and Chittajallu [19] utilized a single-phase two-dimensional electrochemical model, coupled with a nonlinear constrained optimization algorithm, which was solved using sequential quadratic programming (SQP) to obtain the operational and geometric parameters for achieving the maximum electric current in a PEMFC. The parameters investigated include air inlet pressures and cathode thickness, cathode length for each shoulder segment of flow channel, and a fraction of cathode length associated with the flow channel. Similarly, Na and Gou [20] used SQP to optimize the fuel cell system efficiency and cost. Guo et al. [21] proposed an optimization algorithm that combines the teaching–learning based optimization (TLBO) with a differential evolution (DE) algorithm, known as the TLBO-DE method, to promote the efficiency of PEMFC. Behrou et al. [22] demonstrated the use of density-based topology optimization for the practical design of flow fields for PEMFCs, with goals to maximize both the output power and homogeneity of the current density distribution, as well as permit reduced costs and higher durability. The response surface methodology (RSM) has been employed by Kanani et al. [23] and Xuan et al. [24] to maximize the performance of a PEMFC system. Recently, a comprehensive evaluation of different optimization scenarios for a PEMFC is provided by Sohani et al. [25].

Optimization of controlling parameters at the fuel cell system level, like membrane thickness, size of cathode catalyst particle, and protonic conductivity coefficient of the membrane, however, have been very limited. This can be attributed to the fact that they cannot be changed during the cell utilization [4], which makes it tedious and uneconomical to perform these studies experimentally. Moreover, the complex structure of MEA, comprising of polymer electrolyte membrane sandwiched by agglomerate catalyst layers at both the anode and cathode, adds complexity for the design of experiments due to a multiscale nature of the system. In addition, none of the research studies had focused on the membrane electrode assembly coupled with agglomerate catalyst layer parameters. This paper aims to develop a numerical model to simulate a polymer electrolyte membrane fuel cell with detailed multiscale MEA with an agglomerate catalyst layer model, and determine the optimum values of the previously mentioned parameters that provide maximum current density for various voltage values in the ideal range of operation. The study focuses on sensitivity analysis of design parameters of the membrane electrode assembly, including membrane thickness, membrane equivalent weight, the radius of the cathode catalyst particle, catalyst ionomer resistance, cathode catalyst porosity, a membrane protonic conductivity coefficient, a cathode catalyst hydrophobic angle, ionomer tortuosity, and a cathode catalyst volume fraction. The parameters which have significant impact on the current density magnitude are considered. Once the model is validated, it is used to carry out parameter optimization for better cell performance. In the optimization, the response surface methodology is employed for meta-modelling. RSM is a collection of statistical and mathematical methods for optimizing and predicting responses with limited experimental data at various input factors, as well as performing sensitivity analysis [23]. It is extensively used in the industrial world, particularly in situations where the output is swayed by several input parameters. This method has been widely used in different fields and applications such as metals removal [26], chemical extraction [27,28], and the chemical and environmental engineering field [29,30,31]. As compared to other methods, RSM being a collection of mathematical and statistical techniques, gives a better understanding into the role of different parameters at play and generates a continuous model for visualizing the effect of parameters on the entire range as opposed to the average value of the response [32]. This study pioneers the sensitivity analysis of parameters of MEA coupled with agglomerate catalyst layers using the design of the experiment RSM method, along with the validated three-dimensional numerical model. The total numbers of simulations for combinations of 10 parameters would have been computationally very expensive and could not have been done using traditional parametric studies. The RSM method reduces the number of simulations significantly.

## 2. Methodology

### 2.1. Mathematical Formulation

**Figure 1:**
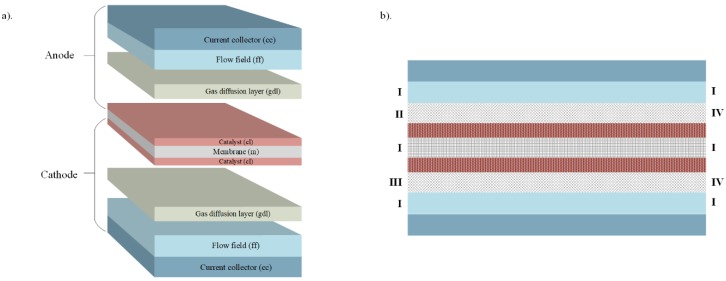
Schematic view of a single PEMFC: (**a**) components of PEMFC, (**b**) computational domains with boundaries: I—insulation/wall, II—anode inlet, III—cathode inlet, and IV—outlets.

The schematic figure of a typical PEMFC and its functional layers is illustrated in Figure 1. The system consists of a proton exchange membrane (m), sandwiched between two catalyst layers (cl), two gas diffusion layers (gdl), two porous metal foam flowfields (ff), and two terminal plates (tp). The main assumptions/approximations adopted in the model are:

- Thermal equilibrium: Local thermal equilibrium between all the phases.
- Membrane: The membrane model takes into account the water flux due to electro-osmatic drag and diffusion.
- Catalyst layers: A cathode particle/agglomerate model is implemented to account for the mass transfer inside the cathode catalyst layer. It is assumed that the particle is spherical and covered by a thin layer of ionomer and water film [33,34,35,36]. The Butler-Volmer equation is employed to calculate the volumetric current transfer or exchange current density.

The mathematical model is comprised of governing equations for the conservation of mass, momentum, species, energy, charge, and water transport in the membrane. The physical parameters, geometry, and operating conditions for two different PEMFC experimental data sets that are used later for validation purposes can be found in Table 1 and Table 2.

#### 2.1.1. Governing Equations

Conservation of Mass [34]:

(1) $$\frac{\partial \rho }{\partial t}+\;𝛻\;\cdot \left(\rho \;\vec{v}\right)=S_{m}$$

where $S_{m}$ is the mass source added from the continuous phase to the dispersed second phase and any other user-defined sources.

Conservation of Momentum [34]:

(2) $$\frac{\partial }{\partial t}\left(\rho \;\vec{v}\right)+𝛻\cdot \;\left(\rho \;\vec{v}\;\vec{v}\;\right)=\;-𝛻\;p+𝛻\cdot \left(\overset{=}{\tau }\right)+\rho \vec{g}+S_{mom}$$

where p denotes pressure, $\overset{=}{\tau }$ is the stress tensor, $\rho \vec{g}$ denotes the gravitational body forces, and $S_{mom}$ is the momentum source term for porous media, which includes the gas diffusion layer, catalyst layer, and membrane. The stress tensor $\overset{=}{\tau }$ is given by the equation below [34].

(3) $$\overset{=}{\tau }=\mu \left[\left(𝛻\;\vec{v}+𝛻\;{\vec{v}}^{T}\right)\;-\;\frac{2}{3}\;𝛻\;\cdot \vec{v}\;I\;\right]$$

where μ is the molecular viscosity and I is the unit tensor. The second term in the right-hand side of the equation represents the effects of volume dilation. 

Species Transport [34]:

(4) $$\frac{\partial }{\partial t}\left(\rho \;Y_{i}\right)+\;𝛻\cdot \left(\rho \;\vec{v}\;Y_{i}\;\right)=\;-𝛻\cdot D_{i,eff}\nabla Y_{i}+S_{m}$$

where $Y_{i}$ denotes the local mass fraction of species i and $D_{i,eff}$ is the effective diffusivity of the species. Note that the total species mixture should conserve the total mass, and, thus, the source terms in the conservation of mass should be equal to the source terms in the conservation of species [48].

Electric Potential [34]:

(5) 𝛻 ·(σ 𝛻 ψ)+S=0

where ψ is the electric potential, σ is the electric conductivity in a solid zone or ionic conductivity in a fluid zone, and S is a source term. 

Conservation of Energy [34]:

(6) $$\frac{\partial }{\partial t}\left(\rho \;E\right)+𝛻\cdot \left(\rho C_{p}\vec{v}T\right)=𝛻\cdot \left(k_{eff}\;𝛻T\right)+S_{h}$$

where $C_{p}$ is the specific heat capacity and $k_{eff}$ is the effective thermal conductivity. The first term on the right-hand side of the equation represents the energy transfer due to conduction.

Volumetric source terms ($S_{m}$) for $H_{2}$ and $O_{2}$ and the dissolved water content λ in the triple-phase boundaries (catalyst layers) due to electrochemical reactions are shown below [34].

(7) $$S_{H_{2}}=\;-\frac{M_{wH_{2}}}{2F}R_{an}<0$$

(8) $$S_{O_{2}}=\;-\frac{M_{wO_{2}}}{2F}R_{cat}<0$$

(9) $$S_{\lambda }=\;-\frac{M_{wH_{2}O}}{2F}R_{cat}>0$$

where $M_{wH_{2}}$, $M_{wH_{2}O}$, and $M_{wH_{2}}$ are the molecular mass of hydrogen, oxygen, and water, respectively, and F is the Faraday constant.

Mass transfer and water transport occurring in the PEMFC model is considered to be in two different phases, which are discussed below.

1. Dissolved phase

The dissolved phase exists in the catalyst layers (ionomers) and in the membrane. The generation and transport of the dissolved water is described by the equation below [49].

(10) $$\frac{\partial }{\partial t}\left(\epsilon_{i}M_{w,H_{2}O}\frac{\rho_{i}}{EW}\;\lambda \right)+\;𝛻\cdot \;\left({\vec{ı}}_{m}\;\frac{\eta_{d}}{F}\;M_{w}\right)=𝛻\cdot \left(M_{w}D^{i}{}_{w}\;𝛻\;\lambda \right)+\;S_{\lambda }+S_{gd}+S_{ld}$$

where $\epsilon_{i}$ denotes the porous media porosity, λ denotes the dissolved water content, η is the osmotic drag coefficient, and ${\vec{ı}}_{m}$ is the ionic current density, calculated as ${\vec{ı}}_{m}=-\beta_{m}\sigma_{mem}\;𝛻\;\phi_{mem}$. In the right hand-side of the equation, $D^{i}{}_{w}$ represents the diffusion coefficient of the water content, $S_{\lambda }$ denotes the water generation rate due to the cathode side reaction in the catalyst layer, $S_{gd}$ is the rate of mass change between gas and dissolved phases, and $S_{ld}$ is the rate of mass change between the liquid and the dissolved phases. The mass change rates are shown in the equations below [50].

(11) $$S_{gd}=\left(1-s^{\theta }\;\right)\;\gamma_{gd}M_{w,H_{2}O\;}\frac{\rho_{i}}{EW}\left(\lambda_{eq}-\lambda \right)$$

(12) $$S_{ld}=\left(s^{\theta }\;\right)\gamma_{ld}M_{w,H_{2}O\;}\frac{\rho_{i}}{EW}\left(\lambda_{eq}-\lambda \right)$$

where $\rho_{i}$ is the dry ionomer or membrane density, EW is the equivalent weight of the membrane, s denotes the liquid saturation, $\lambda_{eq}$ denotes the equilibrium water content, θ is the exponential liquid coverage, and $\gamma_{gd}$, $\gamma_{ld}$ are the gas and liquid mass exchange rate constants and are user-specified parameters. The equilibrium water content $\lambda_{eq}$ can be calculated using the equation below [50].

(13) $$\begin{array}{c} \lambda_{eq}=\;0.3+6a\left(1-tanh\left(a-0.5\right)\right)+0.69\left(\;\lambda_{a=1}-3.52\right)a^{0.5}\left(1+\tanh\left(\frac{a-0.89}{0.23}\right)\right)\\ +s.\left(\lambda_{s=1}-\lambda_{a=1}\right) \end{array}$$

where a is the water activity, defined as:

(14) $$a=\frac{p_{wv}}{p_{sat}}\;$$

where $p_{wv}$ is the water vapour partial pressure and $p_{sat}$ is the saturation pressure. Both $\lambda_{s=1}$ and $\lambda_{a=1}$ in Equation (13) are user-specified parameters.

2. Liquid Phase

Liquid is present in all the porous electrodes and gas channels. The driving force of the liquid water transport is the liquid pressure gradient $𝛻p_{l}$, as shown in the liquid water transport equation below [50].

(15) $$\frac{\partial }{\partial t}\left(\epsilon_{i}\rho_{l}\;s\right)=\;𝛻\;\cdot \left(\frac{\rho_{l}KK_{r}}{\mu_{l}}\;𝛻p_{l}\;\right)+\;S_{gl}-\;S_{ld}$$

In Equation (15), $\rho_{l}$ is the liquid water density, $\mu_{l}$ is the liquid dynamic viscosity, K is the absolute permeability, $K_{r}$ is the relative permeability, $p_{l}$ is liquid pressure, and *S_gl_* is the rate of mass change between the gas and liquid phases. Replacing the liquid pressure with the sum of capillary pressure $p_{c}$ and gas pressure p, Equation (15) can be written as the equation below.

(16) $$\frac{\partial }{\partial t}\left(\epsilon_{i}\rho_{l}\;s\right)=\;𝛻\;\cdot \left(\frac{\rho_{l}KK_{r}}{\mu_{l}}\;𝛻\left(p_{c}+p\right)\right)+\;S_{gl}-\;S_{ld}$$

The mass transfer rate between the gas and liquid phases can be calculated based on the unidirectional diffusion theory [50,51].

(17) $$S_{gl}\{\begin{array}{c} \gamma_{gl}\;\epsilon \;sD_{gl}\frac{M_{w,H_{2}O}}{RT}pln\left(\frac{p-p_{sat}}{p-p_{wv}}\right),\;p_{wv}\leq p_{sat}\;\\ \gamma_{gl}\;\epsilon \;\left(1-s\right)D_{gl}\frac{M_{w,H_{2}O}}{RT}pln\left(\frac{p-p_{sat}}{p-p_{wv}}\right),\;p_{wv}>p_{sat} \end{array}$$

where ϵ is porosity, $\gamma_{gl}$ is the geometric factor of the droplet size, and $D_{gl}\;\left[m^{2}/s\right]$, in the function of temperature [K] and pressure [Pa], takes the following form.

(18) $$D_{gl}\{\begin{array}{c} \;0.365\cdot 10^{-4}{\left(\frac{T}{343}\right)}^{2.334}\cdot \left(\frac{10^{5}}{p}\right),\;cathode\;\\ 1.79\cdot 10^{-4}{\left(\frac{T}{343}\right)}^{2.334}\cdot \left(\frac{10^{5}}{p}\right),\;anode \end{array}$$

Equation (16) is solved in all the regions from the anode GDL-channel interface to the cathode GDL-channel interface. At both interface boundaries, the liquid water flux is considered to leave the gas diffusion layers into the gas channel only. The flux is assumed to be driven by the capillary pressure [50].

(19) $$f_{liq}=max\left[\Theta \epsilon \;sp_{c},0\right]$$

where Θ is the coefficient of liquid water removal. 

Liquid saturation in the channels is calculated from the Leverett function.

(20) $$p_{c}\{\begin{array}{c} \;\sigma \;cos\theta_{c}\;\sqrt{\frac{\epsilon }{K}}\;J\;\left(1-s\right),\;\theta_{c}<90^{{}^{\circ}}\;\\ \sigma \;cos\theta_{c}\;\sqrt{\frac{\epsilon }{K}}\;J\;s,\;\theta_{c}>90^{{}^{\circ}} \end{array}$$

where

(21) $$J\left(x\right)=1.417x-2.12x^{2}+1.263\;x^{3}$$

Liquid water transport in the gas channels is determined to predict the pressure drop increase using the following correlation.

(22) $$\frac{\partial }{\partial t}\left(\rho_{l}\;s\right)\;+𝛻\;\cdot \left(p_{l}{\vec{v}}_{l}s\;\right)=\;𝛻\cdot \left(D_{liq}\;𝛻s\right)$$

where $D_{liq}$ is the liquid water diffusion coefficient in the gas channel and the liquid velocity ${\vec{v}}_{l}$ is assumed to be a fraction of the gas velocity ${\vec{v}}_{g}$, i.e., ${\vec{v}}_{l}=\chi {\vec{v}}_{g}$.

Since the flow-channels (ff) in our model are porous in nature, user-defined function (UDF) is used to add the corresponding source term to X, Y, and Z momentum for the inertial losses.

(23) $$S_{i}=-\left(\frac{\mu }{\alpha }\;v_{i}+\;C_{2}\;\frac{1}{2}\;\rho \;\left|v\right|\;v_{i}\right)$$

where $S_{i}$ denotes the source for the ith (x, y, or z) momentum equation, |v| denotes the magnitude of the velocity, α is the permeability, and $C_{2}$ is the inertial resistance factor.

In laminar flows through porous media, the pressure drop is typically proportional to the velocity and the constant $C_{2}$ can be considered zero. Ignoring the convective acceleration and diffusion, the porous media model is reduced to Darcy’s law.

(24) $$S_{i}=-\;\frac{\mu \;}{\alpha }\;v_{i}$$

The volumetric heat sources in various zones can be found in Table 3, where the variables $i_{s}$ and $i_{m}$ represent the magnitudes of the solid phase and membrane phase current density, respectively, and L (<0) is the latent heat due to water condensation.

#### 2.1.2. Electrochemistry and Cathode Particle/Agglomerate Model

The driving force of these reactions is the surface overpotential, which is the difference between the phase potential of the solid and of the electrolyte or membrane. The phenomenon is accounted for in two equations: one for the electron transport in the catalyst layer, solid grids of porous media, and the current collector, and the other for the protonic conduction or transport of H^+^ at the catalyst and the membrane [52,53].

(25) $$𝛻\cdot \left(\sigma_{sol}\;𝛻\;\phi_{sol}\right)+R_{sol}=0$$

(26) $$𝛻\cdot \left(\beta_{m}\sigma_{mem}\;𝛻\;\phi_{mem}\right)+R_{mem}=0$$

where σ denotes the electrical conductivity in ohm-m^−1^, ϕ denotes the electric potential in volts, and *R* denotes the volumetric transfer current in A·m^−3^, which is also known as exchange current density, expressed as:

(27) $$R_{an}=\left(\;\zeta_{an}\;j_{an}\;\left(T\right)\right){\left(\frac{\left[A\right]}{{\left[A\right]}_{ref}}\right)}^{\gamma_{an}}\;\left(e^{\alpha_{an}^{an}F\eta_{an}/RT}-\;e^{-\alpha_{cat}^{an}F\eta_{an}/RT}\right)$$

(28) $$R_{cat}=\left(\;\zeta_{cat}\;j_{cat}\;\left(T\right)\right){\left(\frac{\left[C\right]}{{\left[C\right]}_{ref}}\right)}^{\gamma_{cat}}\;\left(-e^{\alpha_{an}^{cat}F\eta_{cat}/RT}+\;e^{-\alpha_{cat}^{cat}F\eta_{cat}/RT}\right)$$

In the above equations, $j_{\;}\left(T\right)$ is the reference exchange current density per active surface area [A·m^−2^], ζ is the specific active surface area [m^−1^], [ ] and ${\left[\;\right]}_{ref}$ are the species local concentration and its reference value [kmol·m^−3^], γ is the concentration dependence, $\alpha_{an\;}^{an}$ and $\alpha_{cat\;}^{an}$ are anode and cathode dimensionless transfer coefficients of the anode electrode, respectively, $\alpha_{an\;}^{cat}$ and $\alpha_{cat\;}^{cat}$ are the anode and cathode dimensionless transfer coefficients of cathode electrode, $\eta_{an}$ is the surface overpotential, F is the Faraday constant (9.65 × 10^7^ C·kmol^−1^), R is the universal gas constant, and T is the temperature. 

The reference exchange current densities $j_{an}\left(T\right)$ and $j_{cat}\left(T\right)$ are dependent on a local temperature, described as follows [52,53].

(29) $$j_{an}\left(T\right)=j_{an}^{ref}\;e^{-E_{an}/RT\left(1-T/T_{an}^{ref}\right)}$$

(30) $$j_{cat}\left(T\right)=j_{cat}^{ref}\;e^{-E_{cat}/RT\left(1-T/T_{cat}^{ref}\right)}\;$$

where E and $T_{ref}$ are user-specified activation energy and reference temperature, respectively, and $j_{an}^{ref}$ and $j_{cat}^{ref}$ are the associated exchange current densities at the specified reference temperature. 

The driving force for the kinetics is the local surface overpotential η, also known as the activation loss. It is defined as the difference between the solid and membrane potentials, $\phi_{sol}$ and $\phi_{m}$.

(31) $$\eta_{an}=\phi_{sol}-\phi_{m}-U_{an}^{0}$$

(32) $$\eta_{cat}=\phi_{sol}-\phi_{m}-U_{cat}^{0}$$

The half-cell potentials at the cathode and anode, $U_{an}^{0}$ and $U_{cat}^{0}$, can be calculated using the Nernst equation [50].

(33) $$U_{an}^{0}=E_{an}^{0}-\;\frac{\Delta S_{an}}{2F}\left(T-T^{0}\right)-\frac{RT}{2F}ln\frac{p_{H_{2}}}{p^{0}}$$

(34) $$U_{cat}^{0}=E_{cat}^{0}-\;\frac{\Delta S_{cat}}{2F}\left(T-T^{0}\right)-\frac{RT}{2F}ln\frac{p_{H_{2}o}}{p_{sat}\sqrt{p_{o_{2}}/p^{0}}}$$

where $E^{0}$ denotes the reversible potential, ΔS denotes the reaction entropy, $p_{sat}$ denotes the saturation pressure of water, $T^{0}$ and $p^{0}$ are standard temperature and pressure, and $p_{H_{2}}$, $p_{o_{2}}$, and $p_{H_{2}o}$ are the partial pressures of hydrogen, oxygen, and water vapor, respectively. 

In computing the cathode transfer current using Equation (28), the mass transport resistance of the microstructure is not taken into account in the equation [50]. The resistance consists of two parts, which includes the resistance due to ionomer film $R_{ion}$ and the resistance due to liquid water film surrounding the particles $R_{liq}$. The volumetric transfer current in the cathode layer is represented by the formula below.

(35) $$R_{cat}=4F\frac{c_{O_{2}}}{c_{O_{2}}/j_{O_{2}}^{ideal}+R_{ion}+R_{liq}}$$

where $c_{O_{2}}$ is the oxygen concentration at the wall, $R_{ion}$ is a user-specified value, and $R_{liq}$ can be calculated as the equation below.

(36) $$R_{liq}=\frac{\zeta_{cat}r_{p}^{2}}{K_{w}D_{w}}\;\cdot \;\frac{\sqrt[{3}]{1+\frac{s\epsilon }{1-\epsilon }}}{3\left(1-\epsilon \right)}$$

where $\zeta_{cat}$ is the specific active surface area for the cathode catalyst [m^−1^], s is the liquid saturation, ϵ is the porosity, $r_{p}$ is the particle diameter, and $K_{w}D_{w}$ is the product of oxygen solubility and diffusivity in liquid water. 

The parameter $j_{O_{2}}^{ideal}$ in Equation (35) is defined as the equation below.

(37) $$j_{O_{2}}^{ideal}=\frac{R_{cat}^{0}}{4F}$$

In this case, $R_{cat}^{0}$ is the ideal current transfer, computed using Equation (28) without considering the resistances. 

#### 2.1.3. Constitutive Relations

The density of the gas is given by the equation below.

$$\rho =\;\frac{p\;M}{R\;T}$$

where the mixture molecular weight, expressed in terms of molar fraction of individual species $x_{i},$ is given by the equation below.

$$M=M_{O_{2}}x_{O_{2}}+M_{H_{2}}x_{H_{2}}+M_{H_{2}O}x_{H_{2}O}+M_{N_{2}}x_{N_{2}}$$

The molar fractions are related to the mass fractions shown below.

$$\omega_{i}=\frac{M_{i}x_{i}}{M}$$

Molar concentration of species i is defined as:

$$c_{i}=\frac{P}{RT}\;\times x_{i}$$

and can be calculated as:

$$c_{i}=x_{i}\left(c_{O_{2}}+c_{O_{2}}+c_{H_{2}O}+c_{N_{2}}\right)$$

The relative humidity percentage η is a function of water saturation pressure $p_{H_{2}O}^{sat}$.

$$\eta =\;\frac{px_{H_{2}O}}{p_{H_{2}O}^{sat}\;}\;\times 100$$

$$p_{H_{2}O}^{sat}=101325\;\times 10^{c_{1}+c_{2}\left(T-T_{0}\right)+c_{3}{\left(T-T_{o}\right)}^{2}+c_{4}{\left(T-T_{0}\right)}^{3}}$$

Assuming the ratio $\frac{x_{O_{2}}}{x_{N_{2}}}=\frac{21}{79}$, the oxygen molar fraction at the inlet can be determined from the equation below.

$$x_{O_{2}}^{in}=1-\frac{x_{H_{2}O}^{in}}{1+79/21}$$

while the mole fraction on the anode side is defined below.

$$x_{\;H_{2},a\;}=1-x_{H_{2}O,a}$$

The inlet velocities at the cathode and anode side are given by the following equations, respectively.

$$v_{a}^{in}=\xi_{a}^{in}\frac{i_{avg}\;A_{mem}}{2FA_{inlet}}\times \frac{1}{c_{H_{2}}^{in}}$$

$$v_{c}^{in}=\xi_{c}^{in}\frac{i_{avg}\;A_{mem}}{4FA_{inlet}}\times \frac{1}{c_{O_{2}}^{in}}$$

The average current density $i_{avg}$ is given by the equation below.

$$i_{avg}=\frac{1}{L}\;\int_{o}^{L}i\;\cdot e_{y}\;dx$$

where L is the length of the fuel cell. 

#### 2.1.4. Boundary Conditions

The boundaries of the system as illustrated in Figure 1b are as follows.

- At the side walls:
  $$u^{\left(g\right)}=0,\;\frac{\partial \omega_{i}^{\left(g\right)}}{\partial x}\;=\;\frac{\partial \phi^{\left(s\right)}}{\partial x}=\frac{\partial \phi^{\left(m\right)}}{\partial x}=\frac{\partial s}{\partial x}=\frac{\partial T}{\partial x}=0$$
- At the anode inlet:
  $${\dot{m}}_{a}^{\left(g\right)}={\dot{m}}_{a}^{in}\;,\;\omega_{H_{2}}^{\left(g\right)}=\omega_{H_{2},a}^{in}\;,\;\omega_{H_{2}O}^{\left(g\right)}=\;\omega_{H_{2}O,a}^{in}\;,\;T=T^{in}\;,s=0$$
- At the cathode inlet:
  $${\dot{m}}_{c}^{\left(g\right)}={\dot{m}}_{c}^{in}\;,\;\omega_{H_{2}}^{\left(g\right)}=\omega_{H_{2},c}^{in}\;,\;\omega_{H_{2}O}^{\left(g\right)}=\;\omega_{H_{2}O,c}^{in}\;,\;T=T^{in}\;,s=0$$
- At the outlets:
  $$p^{\left(g\right)}=p^{ref}\;,\;\frac{\partial \omega_{i}^{\left(g\right)}}{\partial x}\;=\;\frac{\partial \phi^{\left(s\right)}}{\partial x}=\frac{\partial s}{\partial x}=\frac{\partial T}{\partial x}=0$$
- At the anode wall terminal:
  $$\phi^{\left(s\right)}=0$$
- At the cathode wall terminal:
  $$-\sigma^{\left(s\right)}\frac{\partial \phi^{\left(s\right)}}{\partial n}=i^{set}$$

The governing equations together with the constitutive relations and appropriate boundary conditions are then solved numerically.

### 2.2. Numerical Method

The developed mathematical model is implemented and customized in the commercial computational software ANSYS Fluent and its PEMFC module together with user-defined functions. The latter allows for changes in constitutive relations, parameters, and—to some extent—the governing equations. Fluent solves all the equations throughout the domain, so variables in the layers that should not be solved are set to zero.

The computational domains (Figure 1b) are created in the commercial software ANSYS Design Modeller and ANSYS Meshing. The whole domain is defined porous, except for the current collectors (cc) and the wall terminals. The whole domain is partitioned into smaller domains for running it in a parallel mode. The computational model is partitioned using the Cartesian Z-direction method to prevent any floating-point exceptions or errors. With convergence criteria of $10^{-6}$ for the residuals of all the conservation equations, iterations are performed, after the mesh independence test to ensure an accurate solution.

### 2.3. Response Surface Methodology

In general concern of a process or system involving a response y that depends on many other controllable input variables $\xi_{1},\;\xi_{2},\;\xi_{3},\;\ldots ,\;\xi_{k}\;,$ its relationship with y is given by the equation below [54].

$$y=f\left(\xi_{1},\xi_{2},\xi_{3},\ldots \xi_{k}\right)+\epsilon$$

where ϵ represents other sources for variability unaccounted for in f. Treating ϵ as a statistical error, we assume it to have a normal distribution with a mean zero and variance $\sigma^{2}$.

### 2.4. Kriging-Based Response Surface Methodology

Design and analysis of computer experiments (DACE) is also called the kriging method of response surface generation. It involves training points in estimating the unknown parameters α and predicting new response points. It interpolates the model at all the training points. This method is used to generate response surfaces for voltage values of 0.6 and 0.8 V. The response can be expressed by the equation below [54].

$$\hat{y}\left(x\right)=f\left(x\right)+z\left(x\right)$$

where f(x) is a low-order polynomial that interpolates the design points. Typically, a constant value is found in order to predict for modelling complex input-output relations. Hence, the output can be viewed as:

$$\hat{y}\left(x\right)=\beta +z\left(x\right)$$

z(x) is a Gaussian stochastic function representing the realization of a random process with zero mean, variance $\sigma^{2},$ and its covariance is given by the equation below.

$$Cov\left(Z\right)=\sigma^{2}R\left(x^{i},x^{j}\right)$$

where $R\left(x^{i},x^{j}\right)$ is the correlation matrix, defined by the equation below.

$$R\left(x^{i},x^{j}\right)=exp\left[-d\left(x^{i},x^{j}\right)\right]$$

$$d\left(x^{i},x^{j}\right)=\;\overset{k}{\underset{l=1}{\sum }}\theta_{l}\left({\left|x_{l}^{i}-x_{l}^{j}\right|}^{p}\right)$$

where i,j denote the two training points, *l* refers to the design parameter, θ is the positive weight factor related to each design parameter, and k denotes the number of design parameters. The mean parameter β is evaluated by the equation below.

$$\beta ={\left[A^{T}\;R^{-1}\;A\right]}^{-1}\;A^{T}\;R^{-1}y$$

where A is an n×n matrix of training set points depending on the choice of the function f(x). The parameters θ and p ensure best fit to the training data. They are evaluated by using a maximizing likelihood estimation (MLE) [55].

$$-\frac{1}{2}\left[n\ln\left(2\pi \right)+n\;ln\sigma^{2}\;+\ln\left|R\right|+\;\frac{1\;}{\;2\sigma^{2}}{\left(y-A\;\beta \right)}^{T}R^{-1}\left(y-A\beta \right)\right]$$

where the maximum likelihood $\sigma^{2}$ is expressed by the equation below.

$$\sigma^{2}=\frac{1}{n}{\left(y-A\beta \right)}^{T}R^{-1}\left(y-A\beta \right)$$

The response at a new point $\bar{x}\;,\;\hat{y}\left(\bar{x}\right)$ is directly evaluated by applying the equation below.

$$\hat{y}\left(\bar{x}\right)=\beta +r^{T}\left(\bar{x}\right)R^{-1}\left(y-A\beta \right)$$

where $r\left(\bar{x}\right)$ is a correlation vector between $\bar{x}$ and all the training points.

## 3. Results and Discussion

### 3.1. Validation

Due to the complexity of the model being solved, model validation with experimental data is imperative to prevent misleading conclusions in predicting the behavior of the fuel cell system. In this work, we aimed to validate our model with two experimental single cells. The first experimental cell was equipped with a Gore Primea 5510 membrane, which is a microscopically reinforced composite membrane. The expressions for various phenomenological membrane models are generally based on the Nafion membrane and so need to be adapted to account for the Gore membrane used in the experiment. Two parameters have been, therefore, adapted to validate the model using both the polarization curve and its iR-corrected counterpart.

The iR-corrected potential is given by the equation below.

$$E_{IR}=E_{rev}-\eta_{a}-\eta_{c}$$

where $\eta_{a}\;\left(>0\right)$ and $\eta_{c}\;\left(<0\right)$ are the corresponding overpotentials of the anode and the cathode catalyst layers, respectively. In this case, we focused on parameter adaption of the cathode reference exchange current density, $j_{cat}^{ref}$, and the cathode transfer coefficient, $\alpha_{cat\;}^{}$, and retained the anode counterparts from the work of Wang and co-workers [39,56].

**Figure 2:**
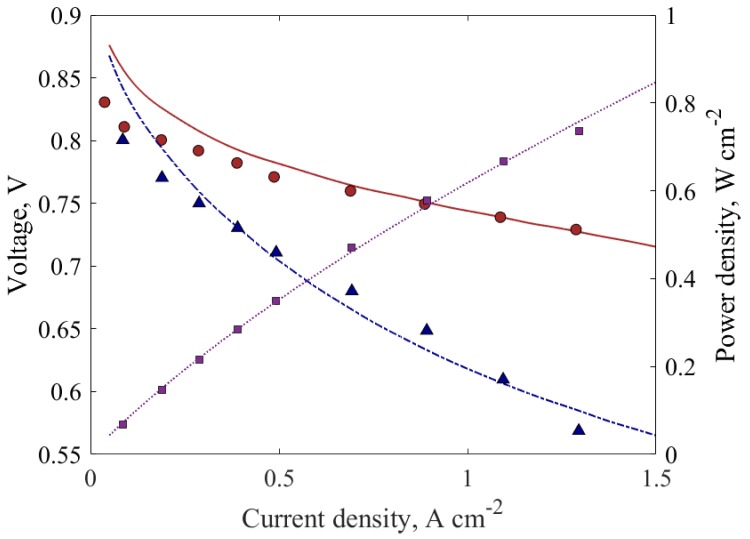
Polarization curves: [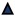] is the experimental potential, [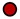] is the iR-corrected experimental potential, [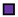] is the experimental power density, [— · ] is the predicted potential, [ — ] is the predicted iR-corrected potential, and [··· ] is the predicted power density.

Two points from the experimentally determined iR-corrected polarization curve were chosen: one at a low current density and the other at a higher current density. Initially, the membrane protonic conductivity coefficient, $\beta_{m}$, was set to one. Once a good agreement for the two points was obtained, we predicted the complete iR-corrected polarization curve. A good agreement for the whole range was achieved, as can be seen in Figure 2. Subsequently, $\beta_{m}$ was varied to finish the validation for the full polarization curve, where the cell voltage is defined below.

$$E_{cell}=E_{rev}-\;\eta_{a}-\eta_{c}-\sum^{\;}i^{\left(s\right)}\;\left(\frac{1}{\sigma_{eff}}\right)\;-\sum^{\;}i^{\left(m\right)}\;\left(\frac{1}{\sigma_{eff}}\right)$$

In this case, the last two terms on the right-hand side of the equation account for the various ohmic losses in the solid and membrane functional layers, respectively.

**Figure 3:**
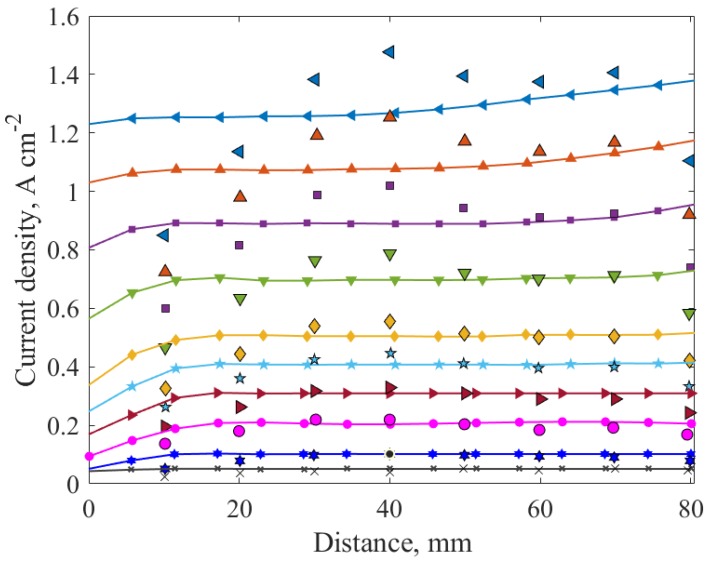
Local current density distribution. Predicted (lines) and experimental (points) for current densities: 1.3 Acm^−2^ (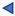), 1.1 A cm^−2^ (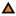), 0.9 A cm^−2^ (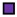), 0.7 A cm^−2^ (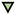), 0.5 A cm^−2^ (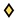), 0.4 A cm^−2^ (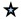), 0.3 A cm^−2^ (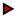), 0.2 A cm^−2^ (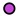), 0.1 A cm^−2^ (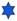), and 0.05 A cm^−2^ (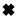).

Furthermore, the predicted local current density distribution along the top of the anode terminal was compared with the experimental counterpart. Figure 3 illustrates this local current density distribution for each measured average current density in Figure 2. It can be observed that better prediction is achieved at lower currents, while the model is less accurate at higher current densities, with the most deviation observed near the inlet and outlet. This could be due to the inlet boundary condition in the simulation that does not represent correctly the position of the inlet manifolds location as in the experiments.

**Figure 4:**
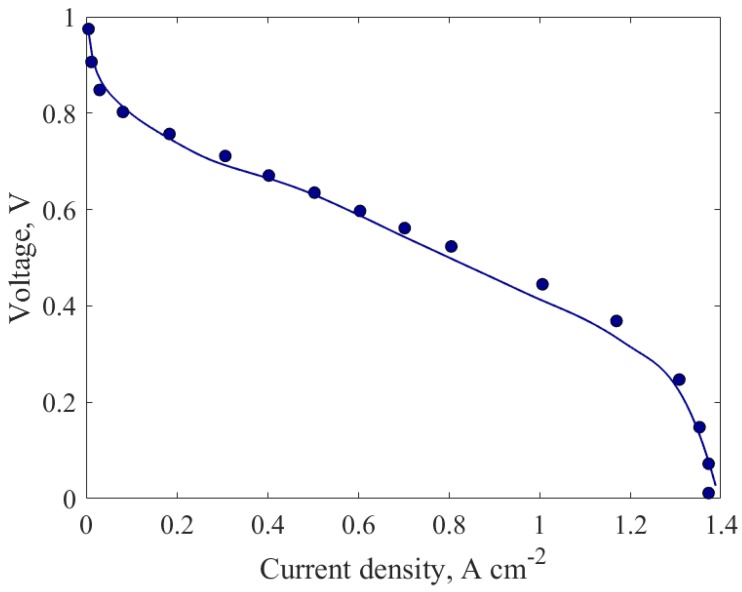
Experimental (points) and predicted (line) polarization curve for case b.

The model is also validated against the second set of experimental polarization curve, especially at a limiting current density to justify the MEA model with an agglomerate catalyst layer. The model was validated with experimental fuel cells with a single-layered gas diffusion studied by Han et al. [37], as depicted in Figure 4. It shows that the model has good agreement with the curve and is able to predict the limiting current density due to mass transport limitations and the presence of two-phase liquid water. 

### 3.2. Response Surface Generation and Optimization

**Figure 5:**
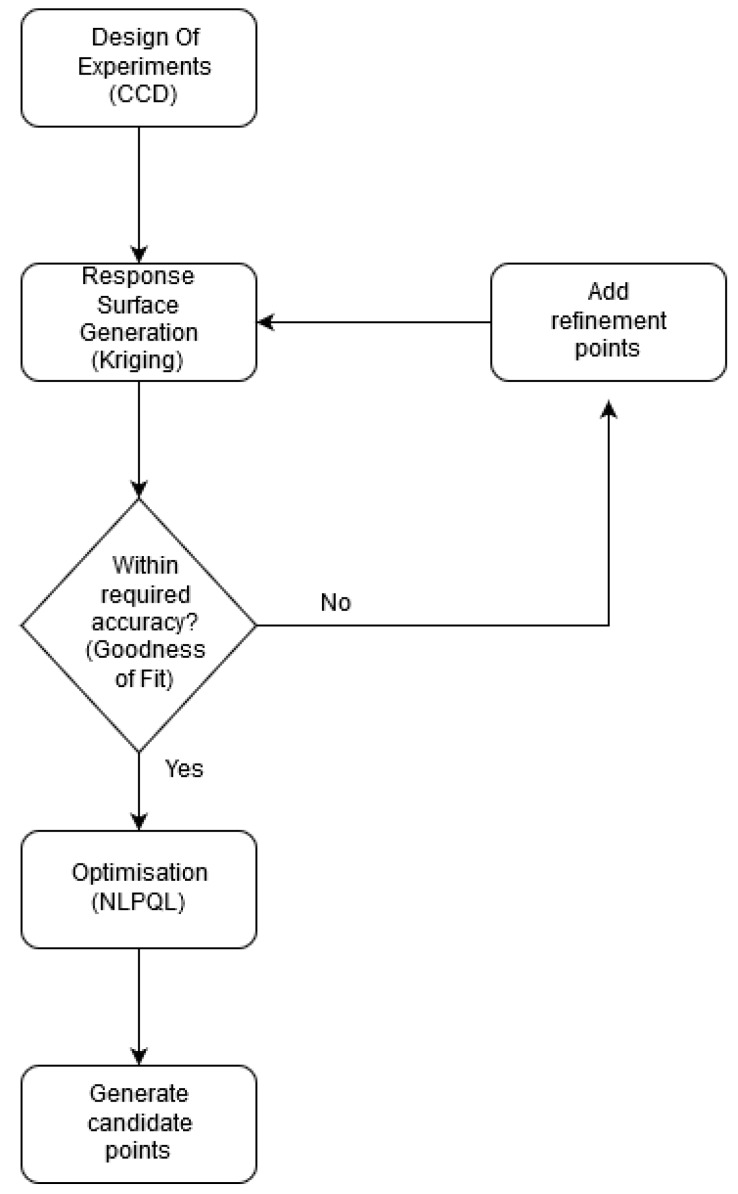
Flow diagram of response surface methodology.

To determine the dominant parameters affecting the PEMFC performance and optimize them, response surface generation and local sensitivity analysis were carried out at medium (0.6 V) and high (0.8 V) voltages. The response surfaces were created using the design of experiments (DOE) method of central composite design (CCD) and the kriging method of response surface generation. The CCD was employed to capture the non-linear interactions that cannot otherwise be described by linear functions. Hence, experimental designs for quadratic response surfaces, like three-level factorial, Box-Behnken, central composite, and Doehlert designs [57], should be used instead. The list of design variables considered for the DOE and their upper and lower bounds are tabulated in Table 4. The base case parameters correspond to case (a) in Table 1, which has been validated for both global and local current densities (Figure 2 and Figure 3). The inputs used for the base-case correspond to a current density of 1 A/cm^2^, i.e., anode stoichiometry of 3.35 ($v_{a}^{in\;}=0.173$ m/s) and cathode stoichiometry of 2.3 $(v_{c}^{in}=\;1.052$ m/s), which are calculated from constitutive relations, as explained in the previous section. The response surface generated was then carefully evaluated. If the response surface was not within the desired limits of accuracy, determined by the “goodness of fit,” it was modified by adding refinement points to the kriging method. Additional CFD simulations were run to generate the response surface data to improve the confidence of the response surface. This iterative method was done until a good fit and reliable response surface was achieved and is illustrated in Figure 5.

**Figure 6:**
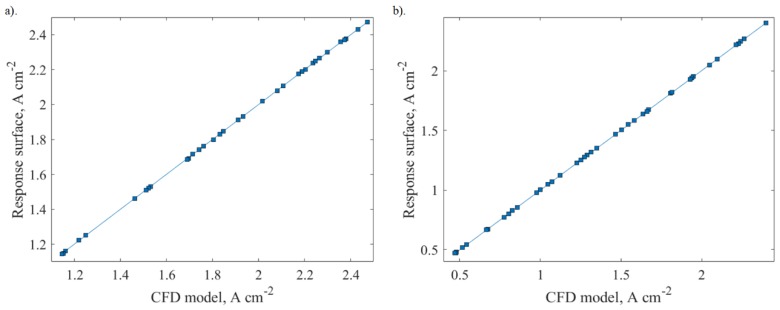
Goodness of fit for response surface generated at: (**a**) 0.8 V and (**b**) 0.6 V.

Goodness of fit was used to determine the reliability of the response surface predicted, i.e., how close the predicted values are to the observed values. The predicted values from the response surface were compared against the values observed from design points for both 0.6 and 0.8 V. The results demonstrate good agreement for both voltage values, which is shown in Figure 6.

### 3.3. Local Sensitivity

In determining which variables influence the output current density the most, the following relation was used.

$$local\;sensitivity=\frac{Output_{max}-Output_{min}}{Output_{average}}$$

**Figure 7:**
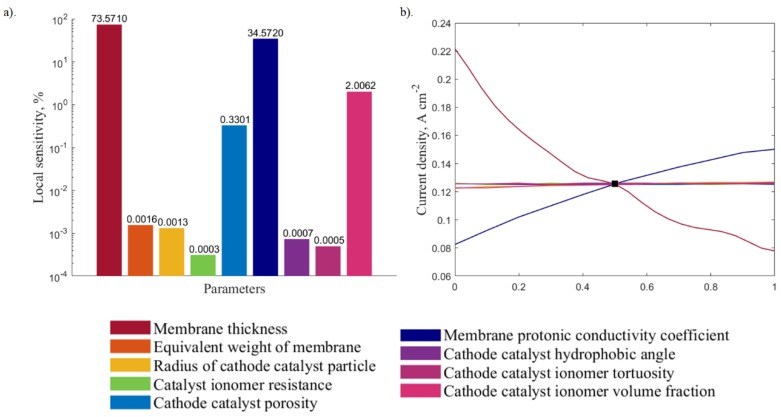
Local sensitivity analysis at 0.6 V presented in: (**a**) bar plot and (**b**) sensitivity curve.

**Figure 8:**
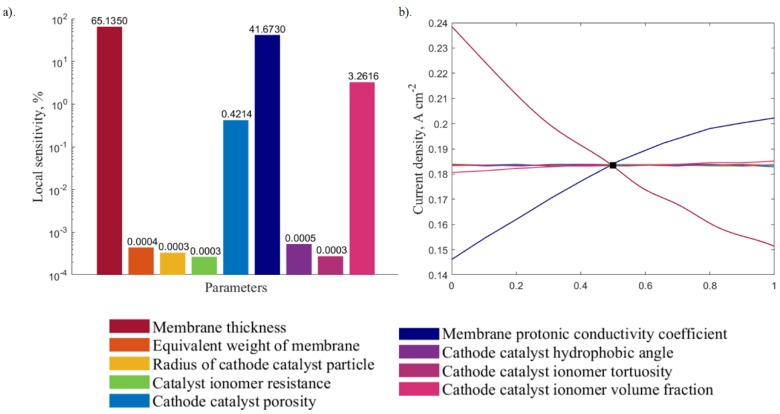
Local sensitivity analysis at 0.8 V presented in: (**a**) the bar plot and the (**b**) sensitivity curve.

The local sensitivity analysis was carried out using outputs obtained from the DOE. Figure 7a and Figure 8a illustrate the relative impacts of the different input parameters on the local sensitivity for the 0.6 and 0.8 V, respectively. The corresponding sensitivity curves (Figure 7b and Figure 8b) show the output variation with changes in one input parameter, while keeping the other parameters constant. The results show that, for both voltage values, parameters that have the most impact are the membrane protonic conductivity coefficient and the membrane thickness. The cathode catalyst ionomer volume fraction, cathode catalyst porosity, cathode catalyst ionomer tortuosity, volume fraction, cathode catalyst hydrophobic angle, and the radius of cathode agglomerate particles have a minor impact on the output that can be considered negligible when compared to these two parameters.

**Figure 9:**
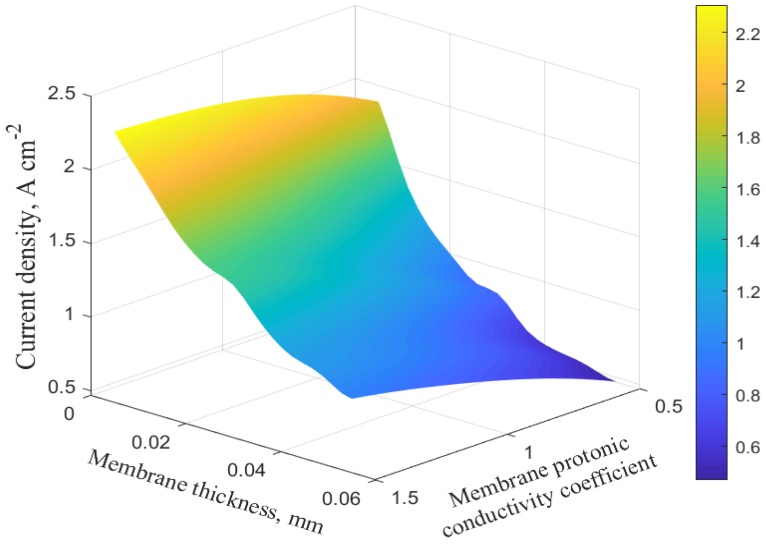
Three-dimensional response surface plot at 0.6 V.

**Figure 10:**
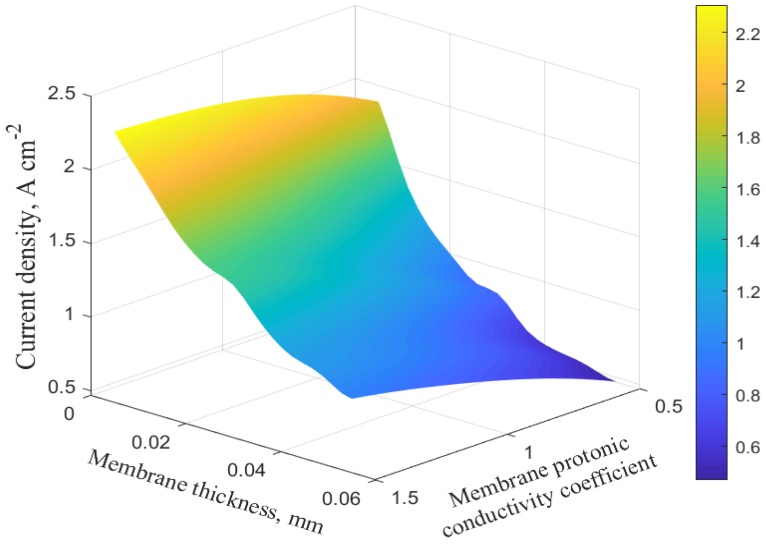
Three-dimensional response surface plot at 0.8 V.

Based on these results, the membrane thickness and the membrane protonic conductivity coefficient are chosen as the varying parameters to study the interrelated response, as well as to perform the optimization. The three-dimensional response charts illustrating how the two variables affect the current output are depicted in Figure 9 and Figure 10 for both medium and high voltages, respectively. These response surfaces are used for the system optimization. 

### 3.4. Optimization

Optimization was carried out using the non-linear programming through a quadratic Lagrangian (NLPQL) approach based on the response surfaces generated previously. NLPQL is a gradient-based algorithm that provides a refined local optimization result. It supports a single constraint on the output parameter and is limited to continuous parameters. The NLPQL approximates derivatives by a central difference scheme and finds candidate points by iterations. This approach was used to determine the optimum values of the variables considered.

Table 5 shows the optimization result that provides maximum current output for three candidate points at 0.8 V. It can be observed that the response surface prediction agrees well with the CFD model, with a maximum error of approximately 0.17%, occurred at candidate point 1 (case i). The current density output increased from 0.18 A cm^−2^ at the base to an average of 0.2472 A cm^−2^, which indicates almost a 40% increase. This increase is attributed to the change in the protonic conductivity coefficient from 0.9 to 1.45 and attributed to the decrease in membrane thickness from 0.03 mm to the lowest bound value of 0.005 mm.

Similarly, a good agreement between the response surface and the computational fluid dynamics (CFD) model is obtained for the medium-voltage case, as shown in Table 6. At 0.6 V, the error is slightly higher, with a maximum error of 0.57%, which occurred at candidate point 1. As in the case at a high voltage value, the protonic conduction coefficient increased from 0.9 to 1.5 m and the membrane thickness reduced to the lowest bound value. However, the current density output for the 0.6 V almost doubled, from 1.23 A cm^−2^ at the base case to 2.4 A cm^−2^, which gives approximately a 96% increase. This large improvement demonstrates the significance of optimization at medium voltage levels, as compared to high voltage values. 

It can be observed that the extent of optimization required increased greatly when the voltage is reduced from a high to a medium level. This is due to the fact that an increase in current density requires better conductivity of the H^+^ ions through the membrane to maintain system net neutrality. In addition, the reduction in membrane thickness decreases the resistance offered by the membrane for the hydrogen ions to move from the anode to the cathode side. The sensitivity analysis also shows that the influence of membrane thickness on the output is greater than that of the protonic conductivity coefficient and rises slightly when the voltage is reduced from 0.8 to 0.6 V. Furthermore, the different values of parameters, other than the two mentioned, in Table 5 and Table 6, confirms the insignificance of these variables to the current output and, hence, varying them would not affect the output largely. 

In short, it can be deduced that, in designing high performance PEMFC, one needs to aim for thinner MEA with higher membrane protonic conductivity, which can be achieved by using carbon-reinforced membrane or water absorbent materials including polytetrafluoroethylene (PTFE), polyvinylidene fluoride (PVDF), and fluorinated ethylene propylene (FEP) or silica gels.

## 4. Conclusions

A numerical study of the two-phase PEMFC with a detailed multiscale agglomerate catalyst layer model was developed and validated against two sets of experimental data for iR-corrected and full polarization curves, including at limiting current densities due to mass transport limitation and the local current density distributions. The model is then extended and coupled with response surface methodology to optimize the design of the membrane electrode assembly (MEA), i.e., membrane thickness, equivalent weight of membrane, radius of agglomerate catalyst particle, cathode catalyst ionomer resistance, porosity of the catalyst layer, a membrane protonic conductivity coefficient, a hydrophobic angle of the catalyst layer, ionomer tortuosity, and a catalyst layer, at high and medium voltages. From sensitivity analysis, it was found that the membrane thickness and membrane protonic conductivity coefficient yield the most significant factor. Reducing the membrane thickness by 40% and increasing protonic conductivity by 50% gives rise to a current density of up to 40% at a higher voltage and up to 100% at a medium voltage. This finding could help fuel cell engineers and designers to carefully manufacture MEA with optimum parameters for a high-performance fuel cell system.

Future work will focus on a combined optimization of design and operating parameters simultaneously for better MEA design, thermal, water, and gas management. A more advanced optimization algorithm including artificial intelligence and machine learning will be considered as well.

## Figures and Tables

**Table 1:** Geometrical and operating parameters for two PEMFC experimental data sets.

	Case a Segmented Cell	Case Single-Gas Diffusion Layer [37]
Physical parameters		
$\kappa_{cl},\kappa_{gdl}$	7.3 × 10^−13^ m^2^	6.1 × 10^−11^ m^2^
$\epsilon_{ff}$	0.9	0.635
$\epsilon_{gdl}$	0.4	0.77
$\sigma_{s,gdl}$	500 S m^−1^	491 S m^−1^
$\sigma_{s,cl}$	500 S m^−1^	491 S m^−1^
$r_{agg}$	10^−7^ m	5 × 10^−6^ m (adapted)
$j_{c,0}^{ref}$	10^3^ A m^−3^ (adapted)	3.5 × 10^4^ A m^−3^ (adapted)
$\alpha_{c}$	2 (adapted)	0.65
$\beta_{m}$	0.9 (adapted)	0.2 (adapted)
Geometry		
$h_{cc}$	5 × 10^−4^ m	6 × 10^−4^ m
$h_{ff}$	5 × 10^−4^ m	6 × 10^−4^ m
$h_{gdl}$	3 × 10^−4^ m	1.1 × 10^−4^ m
$h_{cl}$	10^−5^ m	2 × 10^−5^ m
$h_{m}$	3 × 10^−5^ m	5.1 × 10^−5^ m
L	0.09 m	0.015 m
Operating conditions		
$\eta_{a,c}$	95%, 95%	100%, 100%
$T_{a,c}^{in}$	333 K, 333 K	338 K, 328 K
$p^{ref}$	101,325 Pa	1.5 bars
$\xi_{a,c}$	3.35, 2.3	−
$U_{a,c}^{out}$	−	0.03, 0.16 m s^−1^
$E_{cell}$	0.15−0.85 V	0−0.95 V

**Table 2:** Additional parameters for all cases.

Parameter	Value
$c_{F}$	0.55 [38]
$c_{H_{2}}^{ref}$	40.88 ${\mathrm{mol}\;m}^{-3}$ [39]
$c_{O_{2}}^{ref}$	$\frac{1}{H_{O_{2,\mathrm{pol}}}}{\;\mathrm{mol}\;m}^{-3}$ [40]
$D_{H_{2}}^{0}\;,D_{H_{2}O}^{0}\;,D_{O_{2}}^{0}$	$\left(11.03,\;7.35,\;3.23\right)\;\times 10^{-5}{\;m}^{2}{\;s}^{-1}$ [39]
$D_{O_{2},m}^{0}$	$3.1\;\times 10^{-7}e^{\left(-\;\frac{1768}{T}\right)}\;m^{2}\;s^{-1}$ [40]
$E_{a}$	73,269 ${J\;\mathrm{mol}}^{-1}$ [41]
$E_{rev}^{0}$	1.23 V [39]
F	96,487 ${A\;s\;\mathrm{mol}}^{-1}$
$H_{O_{2},pol},\;H_{O_{2},liq}$	$1.33\;\exp(-666/T),\;5.08\;\exp(-498/T)\;{\mathrm{atm}\;m}^{3}{\;\mathrm{mol}}^{-1}$ [40]
$j_{a,0}^{ref}$	$10^{9}{\;A\;m}^{-3}$ [39]
$k_{cc},k_{cl},\;k_{ff},k_{gdl},k_{m}$	16.3, 1.5, 13.3, 1.5, 0.1 ${W\;m}^{-1}K^{-1}$ [42,43,44,45]
$k_{H_{2}},k_{H_{2}O}\;,\;k_{N_{2}}\;,k_{O_{2}}$	(20.285, 2.16, 2.82, 2.89) × 10^-2^ ${W\;m}^{-1}K^{-1}$ [46]
$M_{H_{2}},M_{H_{2}O}\;,M_{N_{2}}\;,M_{O_{2}}$	$\;\left(2,\;18,\;28,\;32\right)\;\times 10^{-3}{\;\mathrm{kg}\;\mathrm{mol}}^{-1}$
$M_{m}$	1.1 ${\mathrm{kg}\;\mathrm{mol}}^{-1}$ [39]
$m_{pol}$	$10^{-2}{\;\mathrm{kg}\;m}^{-2}$ [37]
R	8.314 ${J\;\mathrm{mol}}^{-1}K^{-1}$
$\alpha_{a}$	1 K [39]
$\gamma_{gd},\gamma_{ld}$	0.5
ϑ	1
$k_{ff},k_{m}$	$10^{-8}\;,\;10^{-18}$$m^{2}$ [47]
μ	1.9 $\times 10^{-5}{\;\mathrm{kg}\;m}^{-1}s^{-1}$ [45]
$\mu_{H_{2}}\;,\mu_{H_{2}O\;},\mu_{N_{2}}\;,\mu_{O_{2}}$	(9.656, 10.98, 19.39, 22.62) $\times 10^{-6}{\;\mathrm{kg}\;m}^{-1}s^{-1}$ [46]
$\rho_{C}\;,\rho_{m}\;,\rho_{Pt}$	(1.8, 2, 21.45) $\times 10^{3}{\;\mathrm{kg}\;m}^{-3}$ [39,40]
$\sigma_{s,cc}\;,\sigma_{s,ff}$	(1.37, 0.1) $\times 10^{6}{\;S\;m}^{-1}$ [42,45]
$c_{1},c_{2}$, $c_{3},c_{4}$	−2.1794, 2.953$\times 10^{-2}$, −9.1837 $\times 10^{-5},\;1.4544\;\times 10^{-7}\;;-,\;K^{-1},\;K^{-2},\;K^{-3}$ [47]
$T_{0}\;,T_{1},\;T_{2}$	273.15, 353.15, 298.15 K [39]

**Table 3:** Energy source terms for the governing equations of conservation energy [50].

Zone	Additional Source Terms
GDL+MPL	$\frac{i_{s}^{2}}{\sigma_{sol}}-S_{gl}\;\cdot L$
Anode catalyst layer	$R_{an}\left(\eta_{an}-\frac{T\Delta S_{an}}{2F}\right)+\frac{i_{s}^{2}}{\sigma_{sol}}+\frac{i_{m}^{2}}{\sigma_{mem}}-\left(S_{dl}+S_{gl}\right)\cdot L$
Cathode catalyst layer	$R_{cat}\left(-\eta_{cat}-\frac{T\Delta S_{cat}}{2F}\right)+\frac{i_{s}^{2}}{\sigma_{sol}}+\frac{i_{m}^{2}}{\sigma_{mem}}-\left(S_{dl}+S_{gl}\right)\cdot L$
Membrane (solid)	$\frac{i_{m}^{2}}{\sigma_{mem}}$
Current collector (solid)	$\frac{i_{s}^{2}}{\sigma_{sol}}$
Gas channels	-

**Table 4:** Design variable values for the DOE method.

Design Variable	Base Case	Lower Bound	Upper Bound
Proton Exchange Membrane thickness	0.03 mm	0.005 mm	0.05 mm
Equivalent weight of membrane	1100 kg kmol^−1^	700 kg kmol^−1^	1500 kg kmol^−1^
Radius of cathode catalyst particle	10^−7^ m	10^−8^ m	10^−7^ m
Cathode Catalyst Ionomer resistance	25 s m^−1^	10 s m^−1^	100 s m^−1^
Porosity of the cathode catalyst layer	0.4	0.2	0.7
Protonic conduction coefficient of the membrane	0.9	0.5	1.5
Hydrophobic angle of the cathode catalyst layer	95°	90°	180°
Ionomer tortuosity of the cathode catalyst layer	1	0.7	1.5
Ionomer volume fraction of the cathode catalyst layer	1	0.5	1.0

**Table 5:** Optimization result for three candidate points at 0.8 V.

Parameter	Range of Values	Case i		Case ii		Case iii	
Membrane thickness	0.005–0.05 mm	0.005 mm		0.005 mm		0.005 mm	
Equivalent weight of membrane	700–1500 kg kmol^−1^	700 kg kmol^−1^		746.65 kg kmol^−1^		824.12 kg kmol^−1^	
Radius of the cathode catalyst particle	10^−8^–10^−7^ m	10^−7^ m		9.4613 × 10^−8^ m		86,287 × 10^−8^ m	
Cathode catalyst ionomer resistance	10–100 s m^−1^	10 s m^−1^		10 s m^−1^		10 s m^−1^	
Porosity of cathode catalyst layer	0.2–0.7	0.7		0.7		0.7	
Protonic conduction Coefficient of the membrane	0.5–1.5	1.4537		1.443		1.4533	
Hydrophobic angle of the cathode catalyst layer	90°–180°	180°		180°		180°	
Ionomer tortuosity	0.7–1.5	0.7		1.0354		1.07	
Ionomer volume fraction	0.5–1.0	1		1		1	
Current density magnitude		Response Surface	Numerical Simulation	Response Surface	Numerical Simulation	Response Surface	Numerical Simulation
		**2477 A m** ^−2^	2472.8 A m^−2^	**2475.2 A m** ^−2^	2471.9 A m^−2^	**2474.6 A m** ^−2^	2472.7 A m^−2^
Error %		0.1698%		0.1335%		0.0768%	
% Increase from base case output (1780.6 A m^-2^)		38.87%		39%		38.87%	

**Table 6:** Optimization result for three candidate points at 0.6 V.

Parameter	Range of Values	Case i		Case ii		Case iii	
Membrane thickness	0.005–0.05 mm	0.005 mm		0.005 mm		0.005 mm	
Equivalent weight of membrane	700–1500 kg kmol^−1^	1500 kg kmol^−1^		1420.1 kg kmol^−1^		1301.1 kg kmol^−1^	
Radius of the cathode catalyst particle	10^−8^–10^−7^ m	10^−8^ m		10^−8^ m		10^−8^ m	
Cathode catalyst ionomer resistance	10–100 s m^−1^	100 s m^−1^		100 s m^−1^		100 s m^−1^	
Porosity of the cathode catalyst layer	0.2–0.7	0.2		0.2		0.2	
Protonic conduction coefficient of the membrane	0.5–1.5	1.5		1.5		1.5	
Hydrophobic angle of the cathode catalyst layer	90°–180°	90°		90°		90°	
Ionomer tortuosity	0.7–1.5	1.5		1.5		1.5	
Ionomer volume fraction	0.5–1.0	1		1		1	
Current density magnitude		Response Surface	Numerical Simulation	Response Surface	Numerical Simulation	Response Surface	Numerical Simulation
		**24166 A m** ^−2^	24029 A m^−2^	**24154 A m** ^−2^	24029 A m^−2^	**24140 A m** ^−2^	24029
Error %		0.57%		0.52%		0.46%	
% Increase from the base case output (12273 A m^−2^)		95.78%		95.78%		95.78%	

## Nomenclature

$A_{cl}$	catalyst area, $m^{2}$
$c_{i}^{\left(g\right)}$	molar concentration of species *i*, mol m^−3^
$C_{i,ref\;}^{\left(g\right)}$	reference molar concentration of species *i*, mol m^−3^
$c_{p}^{\left(g\right)}$	specific heat capacity, J kg^−1^ K^−1^
$c_{1},c_{2},c_{3},c_{4}$	constants for saturation pressure of water, -, K^−1^, K^−2^, K^−3^
$D^{\left(c\right)}$	capillary diffusion, m^2^ s^−1^
$D_{i}$	diffusivity of species *i*, m^2^ s^−1^
$E_{cell}$	cell voltage, V
$E_{a}$	activation energy, J mol^−1^
$E_{rev}$	reversible cell potential, V
F	Faraday constant, C mol^−1^
$h_{j}$	height of layer *j*, m
$H_{O_{2}}^{\left(l\right)},H_{O_{2}}^{\left(p\right)}$	Henry’s constant for air-water and air-polymer interfaces, Pa m^3^ mol^−1^
$\eta_{a,c}$	relative humidity, %
i	current density, A m^−2^
$j_{a,c}^{ref}$	anode and cathode volumetric reference exchange current density, A m^−3^
J	volumetric reference current density, A m^−3^
L	length of channel, m
$m_{H_{2}O}$	interphase mass transport, kg m^−3^ s^−1^
$M^{\;\left(g\right)\;}$	mean molecular mass of the gas phase, kg mol^−1^
$M_{i}$	molecular mass of species, kg mol^−1^
$M^{\left(m\right)}$	equivalent weight of the dry membrane, kg mol^−1^
$n_{d}$	electroosmotic drag coefficient
$p_{H_{2}O}^{sat}$	saturation pressure of water, Pa
R	gas constant, J mol^−1^ K^−1^
s	liquid saturation
S	source term
$T_{0},T_{1},T_{2}$	constants, K
T	temperature, K
V	volume, m^3^
$\omega_{i}^{\left(g\right)}$	mass fraction of species *i*
Greek symbols	
α	transfer coefficient
$\beta_{m}$	membrane modification coefficient
γ	volume fraction
δ	thickness of film, m
ϵ	porosity
η	overpotential, V
θ	wetting angle
κ	permeability, m^2^
λ	membrane water content
μ	dynamic viscosity, kg m^−1^ s^−1^
ξ	stoichiometry
ρ	density, kg m^−3^
τ	surface tension, Pa
σ	total stress tensor, Pa
ϕ	potential, V
Superscripts	
sat	saturation
(g)	gas phase
(m)	membrane
(l)	liquid phase
(s)	solid
in	inlet
ref	reference
(c)	capillary
Subscripts	
α,β	index for species
a	anode
c	cathode
cc	current collector
cl	catalyst layer
ff	flow field
gdl	gas diffusion layer
$H_{2}$	hydrogen
$H_{2}O$	water
i	species i
j	functional layer j
m	membrane
$N_{2}$	nitrogen
$O_{2}$	oxygen
pot	potential
ref	reference
o	standard condition
